# Supplementary material for: Prevalence and associated factors of underweight, overweight and obesity among women of reproductive age group in the Maldives: Evidence from a nationally representative study
Source: PLoS One. 2020 Oct 29;15(10):e0241621. doi: 10.1371/journal.pone.0241621 (PMC7595427; doi:10.1371/journal.pone.0241621)
Supplement: Attachment — Submitted filename: review plose.docx [file pone.0241621.s001.docx]

Comments to authors

I am happy by reviewing this manuscript. For improving the quality of the paper, the following comments were forwarded for the authors.

Title: Prevalence and associated factors of underweight, overweight and obesity among women of the reproductive age group in the Maldives: Evidence from a nationally representative study

1. In the abstract, methods subsection, the author said ‘’Secondary analysis was performed to present descriptive statistics.’’ Make it clear???.
2. In the introduction section; you stated “Watching television was also found to be associated with obesity among reproductive-age women (11)” make it explain in terms of frequency, it is crucial ?? , add more references to this? For support, look at this paper “Mohammed Ahmed, Abdu Seid , and Adnan Kemal. Does the Frequency of Watching Television Matters on Overweight and Obesity among Reproductive Age Women in Ethiopia?. Journal of Obesity. Volume 2020, Article ID 9173075, 7 pages “
3. What makes women different from men in experiencing thus nutritional outcomes. Please state in the introduction appropriately?
4. In the Methods section the author included women of reproductive age ? are you include/ exclude pregnant and postpartum women?
5. In the Methods section, the author states “Overweight and obesity was combined into one category for descriptive purposes.”. Why you merge this outcome in the descriptive?. This is a mistake(misclassification of outcomes)? Therefore, what is the value of the WHO cut off point?
6. In the selection of your variables, Why did not include alcohol drinking status, contraceptive history, and frequency of watching television among the women? This is an important variable
7. Multi-collinearity among the independent variables included in the model was assessed VIF. Does it advisable for a logistic regression analysis?
8. In the descriptive statistics, you reported p-value, from what type of statistic do you get? ( from what type of chi-square, since it is weighted sample due to two-stage stratified cluster sampling)
9. I have a serious concern in your analysis, what type of logistic regression analysis utilized ? is it binary logistic /multinomial logistic?. Besides, How many categories of outcome do you have?
10. Line 184 states “significantly negatively correlated to being underweight compared to the normal weight” reworded it?
11. In the discussion, What is your justification being rural women had a higher prevalence of overweight/obesity in comparison to urban women? Please state it well?
12. Line 284. Harris et al. reported parity as a 285 independent predictor for subsequent maternal weight gain (61). remove Harris et al?
